# Supplementary material for: Identification of immune-related genes in acute myocardial infarction based on integrated bioinformatical methods and experimental verification
Source: PeerJ. 2023 May 16;11:e15058. doi: 10.7717/peerj.15058 (PMC10198157; doi:10.7717/peerj.15058)
Supplement: Supplemental Information 1 — The gene expression of each sample downloaded from public databases. [file peerj-11-15058-s001.zip › Supplemental files 1-2/GO and KEGG.docx]

| ONTOLOGY | ID | Description | GeneRatio | BgRatio | pvalue | p.adjust | qvalue |
| --- | --- | --- | --- | --- | --- | --- | --- |
| BP | GO:0042119 | neutrophil activation | 79/460 | 498/18670 | 2.81e-41 | 1.25e-37 | 9.10e-38 |
| BP | GO:0002283 | neutrophil activation involved in immune response | 78/460 | 488/18670 | 5.44e-41 | 1.25e-37 | 9.10e-38 |
| BP | GO:0043312 | neutrophil degranulation | 77/460 | 485/18670 | 3.06e-40 | 4.69e-37 | 3.41e-37 |
| BP | GO:0002446 | neutrophil mediated immunity | 77/460 | 499/18670 | 2.46e-39 | 2.84e-36 | 2.06e-36 |
| BP | GO:0032496 | response to lipopolysaccharide | 51/460 | 330/18670 | 3.79e-26 | 3.50e-23 | 2.54e-23 |
| CC | GO:0070820 | tertiary granule | 38/476 | 164/19717 | 1.33e-26 | 5.58e-24 | 4.91e-24 |
| CC | GO:0030667 | secretory granule membrane | 42/476 | 298/19717 | 2.02e-20 | 4.24e-18 | 3.74e-18 |
| CC | GO:0042581 | specific granule | 31/476 | 160/19717 | 1.83e-19 | 2.56e-17 | 2.26e-17 |
| CC | GO:0101002 | ficolin-1-rich granule | 32/476 | 185/19717 | 1.65e-18 | 1.73e-16 | 1.53e-16 |
| CC | GO:0070821 | tertiary granule membrane | 20/476 | 73/19717 | 4.02e-16 | 3.38e-14 | 2.98e-14 |
| MF | GO:0038187 | pattern recognition receptor activity | 9/464 | 21/17697 | 1.20e-09 | 8.06e-07 | 7.08e-07 |
| MF | GO:0019865 | immunoglobulin binding | 9/464 | 24/17697 | 5.00e-09 | 1.67e-06 | 1.47e-06 |
| MF | GO:0008329 | signaling pattern recognition receptor activity | 8/464 | 20/17697 | 2.01e-08 | 4.48e-06 | 3.93e-06 |
| MF | GO:0019864 | IgG binding | 6/464 | 11/17697 | 1.30e-07 | 2.17e-05 | 1.91e-05 |
| MF | GO:0005125 | cytokine activity | 21/464 | 220/17697 | 3.63e-07 | 4.85e-05 | 4.26e-05 |
| KEGG | hsa04380 | Osteoclast differentiation | 27/254 | 128/8076 | 1.56e-15 | 4.03e-13 | 3.14e-13 |
| KEGG | hsa04064 | NF-kappa B signaling pathway | 20/254 | 104/8076 | 5.13e-11 | 6.62e-09 | 5.16e-09 |
| KEGG | hsa04668 | TNF signaling pathway | 20/254 | 112/8076 | 2.09e-10 | 1.80e-08 | 1.40e-08 |
| KEGG | hsa05140 | Leishmaniasis | 16/254 | 77/8076 | 1.41e-09 | 9.07e-08 | 7.07e-08 |
| KEGG | hsa04657 | IL-17 signaling pathway | 17/254 | 94/8076 | 4.07e-09 | 2.10e-07 | 1.64e-07 |
