# Supplementary material for: Identification of immune-related genes in acute myocardial infarction based on integrated bioinformatical methods and experimental verification
Source: PeerJ. 2023 May 16;11:e15058. doi: 10.7717/peerj.15058 (PMC10198157; doi:10.7717/peerj.15058)
Supplement: Supplemental Information 4 [file peerj-11-15058-s004.docx]

Codes and packages of R, version 4.0.5,

Work Platform, Rstudio

**Variance analysis using the limma package (Figure 2)**

library(limma)

library(dplyr)

df <- read.table("Counts_data.txt", header = T, sep = "\t", row.names = 1, check.names = F)

head(df)

list <-c(rep("group 1"), rep("group 2")) %>% factor(., levels = c("group 1", "group 2"), ordered = F)

> head(list)

list <- model.matrix(~factor(list)+0)

colnames(list) <-c("group 1", "group 2")

df.fit <-lmFit(df, list)

df.matrix <-makeContrasts(tumor - normal, levels = list)

fit <- contrasts.fit(df.fit, df.matrix)

fit <- eBayes(fit)

tempOutput <- topTable(fit,n = Inf, adjust = "fdr")

**Volcano map (Figure 2AB)**

library(tidyverse)

library(ggplot2)

data <-tempOutput

data <- read.table("~/file.txt", header = T)

head(data)

data$col <- "no significant"

data$col[data$padj < 0.05 & data$logFC > 2] <- "Up"

data$col[data$padj < 0.05 & data$logFC < -2] <- "Down"

data$col <- factor (data$col, levels = c("Down", "no significant","Up"))

data$size <- 1

data$size[data$padj < 0.05 & data$logFC > 2] <- 2

data$size[data$padj < 0.05 & data$logFC < -2] <- 2

ggplot() +

geom_point(data = data, aes(logFC, -log10(padj), colour = col, fill = col),

size = data$size) +

scale_colour_manual(values = c("#4DBBD5", "grey", "#E64B35")) +

geom_vline(xintercept = c(-2, 2), color="grey40", linetype=2) +

geom_hline(yintercept = -log10(0.05), color="grey40", linetype=2)

**Heatmap (Figure 1FG)**

library(tidyverse)

library(ComplexHeatmap)

pheno <- read.table("~/pheno.txt", header = T)

head(pheno)

expr <- read.table("~/expr.txt", header = T, row.names = T)

head(expr[, 1:5])

col = c("#4DBBD5", "#E64B35")

names(col) <- c("group1", "group2")

top <- HeatmapAnnotation(group = pheno$group, col = list(group = col))

Heatmap(as.matrix(t(scale(t(expr)))),

top_annotation = top,

show_column_names = F)

**WGCNA (Figure 3)**

BiocManager::install("WGCNA")

library('WGCNA')

options(stringsAsFactors = FALSE)

femData=read.csv("document.csv")

dim(femData)

names(femData)

datExpr0 = as.data.frame(t(femData[, -c(1:8)]))

names(datExpr0) = femData$substanceBXH

gsg = goodSamplesGenes(datExpr0, verbose = 3);

gsg$allOK

if(!gsg$allOK)

{if(sum(!gsg$goodGenes)>0)

printFlush(paste("Removinggenes:",paste(names(datExpr0)[!gsg$goodGenes], collapse =",")));

if(sum(!gsg$goodSamples)>0)

printFlush(paste("Removingsamples:",paste(rownames(datExpr0)[!gsg$goodSamples], collapse =",")));

datExpr0 = datExpr0[gsg$goodSamples, gsg$goodGenes]}

sampleTree = hclust(dist(datExpr0), method ="average");

sizeGrWindow(12,9) pdf(file="Plots/sampleClustering.pdf",width=12,height=9);

par(cex = 0.6)

par(mar =c(0,4,2,0))

plot(sampleTree, main ="Sample clustering to detectoutliers",sub="", xlab="", cex.lab = 1.5,cex.axis= 1.5, cex.main = 2

powers =c(c(1:10),seq(from = 12, to=20,by=2))

sft = pickSoftThreshold(datExpr, powerVector=powers, verbose=5)

sizeGrWindow(9, 5)

par(mfrow =c(1,2));

cex1 = 0.9;

plot(sft$fitIndices[,1], -sign(sft$fitIndices[,3])*sft$fitIndices[,2],

xlab="SoftThreshold(power)",ylab="ScaleFreeTopologyModelFit,signedR^2",type="n",

main =paste("Scaleindependence"));

text(sft$fitIndices[,1], -sign(sft$fitIndices[,3])*sft$fitIndices[,2],

labels=powers,cex=cex1,col="red");

abline(h=0.90, col="red")

plot(sft$fitIndices[,1], sft$fitIndices[,5],

xlab="SoftThreshold(power)",ylab="MeanConnectivity", type="n",

main =paste("Meanconnectivity"))

text(sft$fitIndices[,1], sft$fitIndices[,5],labels=powers, cex=cex1,col="red")

net=blockwiseModules(datExpr,power=6,

TOMType ="unsigned",

minModuleSize=30,

reassignThreshold=0,

mergeCutHeight=0.25,

numericLabels =TRUE,

pamRespectsDendro=FALSE,

saveTOMs = TRUE,

saveTOMFileBase ="femaleMouseTOM", verbose = 3)

sizeGrWindow(12, 9)

mergedColors = labels2colors(net$colors)

plotDendroAndColors(net$dendrograms[[1]],mergedColors[net$blockGenes[[1]]],"Modulecolors",dendroLabels = FALSE, hang=0.03,addGuide = TRUE, guideHang = 0.05)

moduleLabels = net$colors

moduleColors = labels2colors(net$colors)

MEs = net$MEs;

geneTree = net$dendrograms[[1]];

save(MEs, moduleLabels, moduleColors, geneTree,

file="FemaleLiver-02-networkConstruction-auto.RData")

**GO (Figure 4A-C)**

diff<-read.csv(file="C:/Users/Desktop/data.csv")

library(AnnotationDbi)

library(org.Hs.eg.db)

library(clusterProfiler)

library(dplyr)

library(ggplot2)

gene.df<-bitr(diff$SYMBOL,fromType="SYMBOL",

toType="ENTREZID", OrgDb = org.Hs.eg.db）

gene <- gene.df$ENTREZID

ego_ALL <- enrichGO(gene = gene,

OrgDb=org.Hs.eg.db,

keyType = "ENTREZID",

ont = "ALL",

pAdjustMethod = "BH",

minGSSize = 1,

pvalueCutoff = 0.01,

qvalueCutoff = 0.05,

readable = TRUE)

ego_CC <- enrichGO(gene = gene,

OrgDb=org.Hs.eg.db,

keyType = "ENTREZID",

ont = "CC",

pAdjustMethod = "BH",

minGSSize = 1,

pvalueCutoff = 0.01,

qvalueCutoff = 0.05,

readable = TRUE)

ego_BP <- enrichGO(gene = gene,

OrgDb=org.Hs.eg.db,

keyType = "ENTREZID",

ont = "BP",

pAdjustMethod = "BH",

minGSSize = 1,

pvalueCutoff = 0.01,

qvalueCutoff = 0.05,

readable = TRUE)

ego_MF <- enrichGO(gene = gene,

OrgDb=org.Hs.eg.db,

keyType = "ENTREZID",

ont = "MF",

pAdjustMethod = "BH",

minGSSize = 1,

pvalueCutoff = 0.01,

qvalueCutoff = 0.05,

readable = TRUE)

ego_ALL <- as.data.frame(ego_ALL)

ego_result_BP <- as.data.frame(ego_BP)

ego_result_CC <- as.data.frame(ego_CC)

ego_result_MF <- as.data.frame(ego_MF)

ego <- rbind(ego_result_BP,ego_result_CC,ego_result_MF)

write.csv(ego_ALL,file = "ego_ALL.csv",row.names = T)

write.csv(ego_result_BP,file = "ego_result_BP.csv",row.names = T)

write.csv(ego_result_CC,file = "ego_result_CC.csv",row.names = T)

write.csv(ego_result_MF,file = "ego_result_MF.csv",row.names = T)

write.csv(ego,file = "ego.csv",row.names = T)

ego_result_BP <- as.data.frame(ego_BP)[1:display_number[1], ]

ego_result_CC <- as.data.frame(ego_CC)[1:display_number[2], ]

ego_result_MF <- as.data.frame(ego_MF)[1:display_number[3], ]

go_enrich_df <- data.frame(

ID=c(ego_result_BP$ID,ego_result_CC$ID,ego_result_MF$ID), Description=c(ego_result_BP$Description,ego_result_CC$Description,ego_result_MF$Description),

GeneNumber=c(ego_result_BP$Count,ego_result_CC$Count,ego_result_MF$Count),

type=factor(c(rep("biological process", display_number[1]),

rep("cellular component", display_number[2]),

rep("molecular function", display_number[3])),

levels=c("biological process","cellular component","molecular function" )))

for(i in 1:nrow(go_enrich_df)){

description_splite=strsplit(go_enrich_df$Description[i],split = " ")

description_collapse=paste(description_splite[[1]][1:5],collapse = " ") #

go_enrich_df$Description[i]=description_collapse

go_enrich_df$Description=gsub(pattern="NA","",

go_enrich_df$Description)

go_enrich_df$type_order=factor(rev(as.integer(rownames(go_enrich_df))),labels=rev(go_enrich_df$Description))

COLS <- c("#66C3A5", "#8DA1CB", "#FD8D62")

ggplot(data=go_enrich_df, aes(x=type_order,

y=GeneNumber, fill=type)) +

geom_bar(stat="identity", width=0.8) +

scale_fill_manual(values = COLS) +

coord_flip() +

xlab("GO term") +

ylab("Gene_Number") +

labs(title = "The Most Enriched GO Terms")+

theme_bw()

go_enrich_df$type_order=factor(rev(as.integer(rownames(go_enrich_df))),labels=rev(go_enrich_df$Description))

COLS <- c("#66C3A5", "#8DA1CB", "#FD8D62")

ggplot(data=go_enrich_df, aes(x=type_order,y=GeneNumber, fill=type))+

geom_bar(stat="identity", width=0.8) +

scale_fill_manual(values = COLS) +

theme_bw() +

xlab("GO term") +

ylab("Num of Genes") +

labs(title = "The Most Enriched GO Terms")+

theme(axis.text.x=element_text(face = "bold", color="gray50",angle = 70,vjust = 1, hjust = 1 ))

**KEGG (Figure 4D)**

kk <- enrichKEGG(gene = gene,keyType = "kegg",organism= "human", qvalueCutoff = 0.05, pvalueCutoff= 0.05)

hh <- as.data.frame(kk)！

rownames(hh) <- 1:nrow(hh)

hh$order=factor(rev(as.integer(rownames(hh))),labels = rev(hh$Description))

ggplot(hh,aes(y=order,x=Count,fill=p.adjust))+

geom_bar(stat = "identity",width=0.7)+

scale_fill_gradient(low = "red",high ="blue" )+

labs(title = "KEGG Pathways Enrichment",

x="Gene numbers",

y="Pathways")+

theme(axis.title.x = element_text(face = "bold",size = 16),

axis.title.y = element_text(face = "bold",size = 16),

legend.title = element_text(face = "bold",size = 16))+

theme_bw()

hh<- as.data.frame(kk)

rownames(hh) <- 1:nrow(hh)

hh$order=factor(rev(as.integer(rownames(hh)))

labels=rev(hh$Description))

ggplot(hh,aes(y=order,x=Count))+

geom_point(aes(size=Count,color=-1*p.adjust))+

scale_color_gradient(low="green",high="red")+

labs(color=expression(p.adjust,size="Count"), x="Gene Number",y="Pathways",title="KEGG Pathway Enrichment")+

theme_bw()

**Chord diagram (Figure 5B)**

library（circlize）

chordDiagram(mat)

library(tidyverse)

library(reshape2)

df <- melt(mat) %>% rename('from' = 'Var1','to' = 'Var2')

head(df)

from to value

chordDiagram(df)

chordDiagram(mat, order = c("S2", "S1", "S3", "E4", "E1", "E5", "E2", "E6", "E3"))

circos.clear()

circos.par(gap.after = c(rep(5,nrow(mat)-1),15,rep(5,ncol(mat)-1),15))

chordDiagram(mat)

circos.clear()

circos.par(gap.after = c("S1"=5, "S2"=5, "S3"=15, "E1"=5, "E2"=5,

"E3"=5, "E4"=5, "E5"=5, "E6"=15))

chordDiagram(mat, big.gap=30)

circos.par(start.degree=90, clock.wise=F)

chordDiagram(mat)

col=c(S1="red", S2="green", S3 ="blue",E1 ="black", E2 ="cyan", E3 = "orange", E4="green", E5="lavender", E6 = "grey", E7="light blue")

chordDiagram(mat,grid.col=col,transparency=0.5)

circos.clear()

chordDiagram(mat, grid.col = col, link.lwd = 2, link.lty = 2, link.border = "red")

circos.clear()

chordDiagram(mat, grid.col=col, transparency=0, link.zindex = rank(mat))

**Relevance Heat Map (Figure 6AB)**

library(tidyverse)

library(corrplot)

library(ggplot2)

library(ggcorrplot)

data <- read.table("~/file.txt", header = T)

rownames(data) <- data[,1]

data <- data[,-1]

corrplot(as.matrix(data))

p <- ggcorrplot(data)

**LASSO (Figure 7)**

library(readxl);

library(caret);

library(glmnet);

library(corrplot)

library(Metrics);

library(ggplot2)

data<-read.csv("C:/Users/data.csv",sep = ",")

data_cor <- cor(data)

corrplot.mixed(data_cor,tl.col="black",tl.pos = "d",number.cex = 0.8)

set.seed(123)

d_index <- createDataPartition(data$Outcome,p = 0.7)

train_d <- data[d_index$Resample1,]

test_d <- data[-d_index$Resample1,]

scal <- preProcess(train_d,method = c("center","scale"))

train_ds <- predict(scal,train_d)

test_ds <- predict(scal,test_d)

lambdas <- seq(0,2, length.out = 100)

X <- as.matrix(train_ds[,1:8])

Y <- train_ds[,9]

set.seed(1004)

lasso_model <- cv.glmnet(X,Y,alpha = 1,nlambda = 200,nfolds =3)

plot(lasso_model)

plot(lasso_model$glmnet.fit, "lambda", label = T)

lasso_model$lambda.min

lasso_min <- lasso_model$lambda.min

lasso_best <- glmnet(X,Y,alpha = 1,lambda = lasso_min)

coef(lasso_best)

test_pre <- predict(lasso_best,as.matrix(test_ds[,1:8]))

sprintf("The mean absolute error after standardization is: %f",mae(test_ds$Outcome,test_pre))

test_pre_o <- as.vector(test_pre[,1] * scal$std[9] + scal$mean[9])

sprintf("The mean absolute error before standardization is: %f",mae(test_d$Outcome,test_pre_o))

**Lollipop Chart (Figure 8)**

install.packages("ggpubr")

library(ggpubr)

library(ggplot2)

df<-read.delim("Data.txt")

ggdotchart(df, x=colnames(df)[1],

y=colnames(df)[2],

color=colnames(df)[1],

palette=rainbow(dim(df)[1]),

sorting="none",

c("ascending", "descending", "none"),

add="segments",

c("none", "segment")

dot.size=10,

add.params=list(color="lightgray", size=5),

position=position_dodge(0.1),

label=round(df[[2]]),

font.label=list(color="white",

size=9,

vjust=0.5)
